# Height and crown allometries and their relationship with functional traits: An example from a subtropical wet forest

**DOI:** 10.1002/ece3.9804

**Published:** 2023-02-14

**Authors:** Jie Yang, Nathan G. Swenson

**Affiliations:** ^1^ Key Laboratory of Tropical Forest Ecology, Xishuangbanna Tropical Botanical Garden Chinese Academy of Sciences Kunming China; ^2^ Department of Biological Sciences University of Notre Dame Notre Dame Indiana USA

**Keywords:** allocation, diameter‐crown allometry, diameter‐height allometry, functional ecology, tropical forest

## Abstract

Forest tree communities are largely structured by interactions between phenotypes and their environments. Functional traits have been popularized as providing key insights into plant functional tradeoffs. Similarly, tree crown—stem diameter and tree height—stem diameter allometric relationships are likely to be strongly coordinated with functional trait tradeoff axes. Specifically, species with functional traits indicative of conservative strategies (i.e., dense wood, heavy seeds) should be related to tree architectures that have lower heights and wider crowns for a given stem diameter. For example, shade‐tolerant species in tropical forests are typically characterized as having dense wood, large seeds, and relatively broad crowns at early ontogenetic stages. Here, we focus on 14 dominant dicot tree species in a tropical forest. We utilized hierarchical Bayesian models to characterize species‐specific height and crown size allometric parameters. We sampled from the posterior distributions for these parameters and correlated them with six functional traits. We also characterize the expected height and crown size for a series of reference stem diameters to quantify the relationship between traits and tree architecture across size classes. We find little interspecific variation in allometric slopes, but clear variation in allometric intercepts. Allometeric height intercepts were negatively correlated with wood density and crown size intercepts were positively related to wood density and seed mass and negatively related to leaf percent phosphorus. Thus, interspecific variation in tree architecture is generated by interspecific variation in allometric intercepts and not slopes. These intercepts could be predicted using a handful of functional traits where conservative traits were indicative of trees that are relatively short and have larger crown sizes. This demonstrates a coordination of tropical tree life histories that can be characterized simultaneously with functional traits and tree allometries.

## Graphical abstract

The ontogenetic trajectory of height and crown allocation of tropical trees is an essential indicator of their life histories. Here, we show that this whole plant strategy can be linked to organ‐level plant functional traits leading to insights into how phenotypes are integrated.

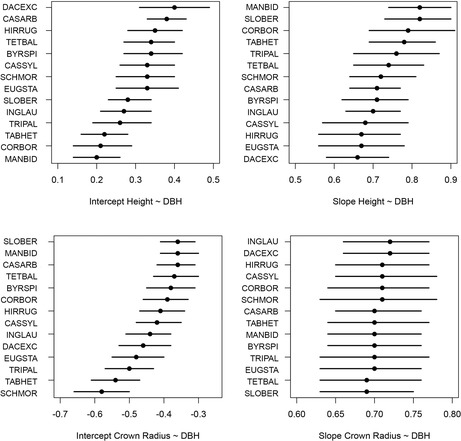

## 1 INTRODUCTION

The structure and dynamics of forest tree communities are the aggregate outcomes of ecological interactions governed by the form and function of species (Swenson, 2012, 2013). This understanding has given rise to a large literature on plant functional traits particularly in plant community ecology (McGill et al., 2006). Plant functional traits are traits that dictate plant demographic performance, and therefore fitness, given an environmental context (Reich et al., 2003). To this end, plant ecologists have generally focused on traits that are indicative of fundamental life‐history tradeoffs either at the individual or organ scale (Chave et al., 2009; Moles & Westoby, 2006; Reich et al., 1997; Westoby et al., 2002; Wright et al., 2004). For example, wood density is a key indicator of the wood economics spectrum where species fall along a continuum of dense wooded species with high survival, but low growth rates (i.e., a conservative strategy) to light wooded species with low survival, but high growth rates (i.e., an acquisitive strategy; Chave et al., 2009).

A key question in community ecology has been the extent to which these fundamental tradeoff axes or trait spectra are coordinated (Díaz et al., 2016; Westoby, 1998; Westoby et al., 2002). In other words, to what extent do species with acquisitive leaf trait strategies also have acquisitive wood and seed trait strategies? If acquisitive species are acquisitive throughout, then it would greatly reduce the dimensionality of niche differences. Conversely, if being acquisitive along one axis (e.g., wood) does not indicate whether a species is acquisitive on another axis (e.g., leaves), then this would greatly increase the dimensionality of plant functional strategies worldwide (Díaz et al., 2016). A related question is: to what degree is whole plant architecture related to the tradeoffs captured by individual traits? One way to approach this problem is to investigate the relationships between tree allometries and functional traits (e.g., Iida et al., 2012).

Tree allometric relationships are often depicted as having universal scaling exponents or parameters (e.g., Niklas, 1994). However, there is notable interspecific variation in whole tree architecture. For example, tree species do vary in their crown size or height for a given stem diameter. Interspecific variation in allometric relationships between tree crown size and stem diameter and tree height and stem diameter is an important indicator of the ecological strategy of trees (Hulshof et al., 2015; Iida et al., 2011, 2012; King, 1990, 1996; Niklas, 1994; Osunkoya et al., 2007; Poorter et al., 2006). For example, when regressing crown size against diameter, the intercept of the scaling relationship indicates the relative crown size given the diameter and the slope indicates the rate of crown size increase with diameter. Thus, larger intercepts indicate that a species has a larger crown at smaller trunk sizes perhaps to facilitate light capture. Trees with relatively larger crowns at small stem diameters may be expected in species that are shade tolerant and maximizing their crown size (e.g., Kitajima, 1994). Therefore, species with higher intercepts for crown size‐stem diameter allometries may have a set of conservative functional trait values (i.e., dense wood, low leaf nutrient content, heavy seed mass). Similarly, a steeper slope indicates a more rapid increase in canopy size with diameter, which may only be facilitated by a mechanically sturdy stem that comes at a high construction cost (i.e., a stem with dense wood; King et al., 2006).

The parameters of height‐stem diameter allometries are also expected to be related to commonly measured plant functional traits. For example, trees that are relatively taller at a given stem diameter are likely on the acquisitive side of the life‐history spectrum. That is, they are likely light‐demanding species that are racing toward the canopy and this is facilitated by cheap stem construction (i.e., light wood) and fast resource acquisition rates. Previous work has indicated that, indeed, wood density does predict tree allometric relationships in tropical rainforest trees (e.g., Iida et al., 2012; King et al., 2006). Additional work in tropical moist forests indicates other functional traits and shade tolerance are related to tree architecture (e.g., Poorter et al., 2006). Specifically, the maximum height of tree species and shade tolerance of tree species have been found to be linked to tree growth trajectories. Despite this previous research, additional work is needed to directly test whether tree allometries and architectures are related to the life‐history tradeoffs captured by commonly measured functional traits. Specifically, we need clear tests of whether conservative functional trait values (i.e., dense wood, heavy seeds, slow leaf economics) are related to trees that are shorter in stature with larger crowns at a given stem diameter. Addressing this would help to improve our understanding of how key functional traits integrate with whole tree architecture to generate an integrative phenotype that is linked to key life‐history tradeoffs.

The present work seeks to address the challenges and predictions above using detailed trait and allometric relationships measured on 14 dominant tree species in the El Yunque National Forest, Puerto Rico. The specific questions we ask are: (i) what allometric parameters are variable among species thereby allowing for diversity in tree architecture? (ii) are functional traits that are related to where a species lands on an acquisitive to conservative ecological strategy related to allometric parameters? and (iii) are the changes in tree architecture (i.e., height and crown size) across trunk diameter sizes related to functional traits?

## 2 METHODS

### 2.1 Study location and species

The present study was conducted in the area around the El Verde Field Station (350 m a.s.l.) located in the El Yunque National Forest on the island of Puerto Rico. The forest is classified as a subtropical wet forest receiving around 3.5 m of rainfall per year. The first 4 months of the calendar year are drier, but no <0.2 m of rain falls during any of these months (Thompson et al., 2004). The forest has been severely impacted in the past by hurricanes including Hugo in 1989 and Georges in 1998 and, most recently, Maria in 2017. The study utilized data collected in 2010, 2015, and 2017 (prior to Hurricane Maria) and all trees measured had no stem breaks and no clear crown damage. The study focused on 14 tree species that are dominate components of the forest ranging in their life‐history strategies (Table 1). The tree community at the elevation studied has roughly 140 species, but the 14 species studied comprise >80% of the dicot stems found in the canopy of the forest. We note that tree dimensions have been measured in detail in the past at this field site (e.g., Odum & Pigeon, 1970), but the data come from a few large individuals and no previous work has linked allometries of these species to their traits.

**TABLE 1:** The 14 species analyzed in this study, their sample sizes (*n*) for allometric measurements.

Family	Binomial	Code	*n*
Malpighiaceae	*Byrsonima spicata*	BYRSPI	10
Salicaceae	*Casearia arborea*	CASARB	17
Salicaceae	*Casearia sylvestris*	CASSYL	10
Boraginaceae	*Cordia borinquensis*	CORBOR	10
Burseraceae	*Dacryodes excelsa*	DACEXC	16
Myrtaceae	*Eugenia stahlii*	EUGSTA	10
Chrysobalanaceae	*Hirtella rugosa*	HIRRUG	10
Fabaceae	*Inga laurina*	INGLAU	25
Sapotaceae	*Manilkara bidentata*	MANBID	17
Araliaceae	*Didymopanax morototoni*	SCHMOR	16
Elaeocarpaceae	*Sloanea beteroana*	SLOBER	25
Bignoniaceae	*Tabebuia heterophylla*	TABHET	10
Burseraceae	*Tetragastris balsamifera*	TEBAL	12
Meliaceae	*Trichilia pallida*	TRIPAL	10

### 2.2 Tree crown and height measurements

For each species, we located at least 10 individuals from a broad range of individual sizes and measured their diameters at breast height (DBH; 130 cm above the ground). Only healthy trees with no evidence of structural damage were chosen and only trees where crowns could be viewed with confidence via binoculars were chosen. After tree diameter measurement, we used binoculars to identify the location of the crown edge, position themselves under that edge and use a laser range finder or tape measure to measure the distance to the tree stem. Next, the canopy edge at 90 degrees from the first measurement was taken using the same methodology. An average of these two measures was used in subsequent analyses. The height of each tree was then measured using the laser range finder. We only utilized trees where there was a clear line of sight to both the base of the trunk and top of the tree.

### 2.3 Functional trait collection

Publicly available functional trait data were collected for the 14 species in this study (Swenson et al., 2011; Swenson, Erickson, et al., 2012; Swenson, Stegen, et al., 2012). Specifically, average trait values have been calculated for eight different traits across all species. The six traits are: leaf %N, leaf %P, wood density (g/cm^3^), leaf area (cm^2^), specific leaf area (SLA; cm^2^/g), and seed mass (g). The leaf %N, leaf %P, and SLA values indicate where species fall along a fast‐to‐slow strategy continuum where species with high values have higher rates of photosynthesis at the cost of shorter leaf lifespans, whereas species with lower values have longer leaf lifespans (Wright et al., 2004). Wood density represents a tradeoff between volumetric growth rates and mortality rates (Chave et al., 2009; Swenson & Enquist, 2007) and is often assumed to be an orthogonal tradeoff access to the leaf tradeoff axis (Baraloto et al., 2010). Seed mass represents a tradeoff between endosperm investment and mortality rates versus growth rates (Venable, 1996). Leaf area is related to leaf temperatures and thermal budgets. All traits were measured using globally standardized collection methods (Cornelissen et al., 2003).

### 2.4 Allometric analyses

In this study, we developed hierarchical models that were evaluated using a Bayesian approach. We fit both the average crown radius and height of an individual tree as a function of the DBH of the tree. The crown radius, height and DBH data were all log_10_ transformed prior to the analyses. The tree height was modeled as:

$$\mu_{i,j}=\alpha_{j}+\beta_{j}*\log_{10}\;{\left(\mathrm{DBH}\right)}_{i,j}$$

where *μ*_*i,j*_ is the expected log_10_ height of individual *i* in species *j*, α_*j*_ and *ß*_*j*_ are the intercept and slope for species *j*, and log_10_ (DBH) is the DBH of individual *i* in species *j*. The average tree crown radius was modeled as:

$$\mu_{i,j}=\alpha_{j}+\beta_{j}*\log_{10}\;{\left(\mathrm{DBH}\right)}_{i,j}$$

where *μ*_*i,j*_ is the expected log_10_ crown radius of individual *i* in species *j*, α_*j*_ and *β*_*j*_ are the intercept and slope for species *j*, and log_10_ (DBH) is the DBH of individual *i* in species *j*. In both models, a covariance between α_*j*_ and *β*_*j*_ parameters was included. The models were evaluated using STAN via the *rethinking* R package (McElreath, 2020; Stan Development Team, 2018). We leveraged global allometries reported for the angiosperms by Niklas (1994) to inform our priors for the *β*_*j*_ parameters. This prior information was used in order to leverage the large amount of allometric knowledge in existence to improve model convergence, particularly where the number of data points within a species was low. Each model was run using four chains with 20,000 iterations and a warmup of 10,000 iterations. A Watanabe–Akaike Information Criterion (WAIC; Watanabe, 2010) was calculated for each model. A WAIC allows for the comparison of multiple Bayesian models while penalizing for parameter number. Tree allometries and height‐diameter allometries, in particular, are typically fit in the literature using either asymptotic or linearized power law functions (e.g., Hulshof et al., 2015). We tested both functions for each allometry and compared them using WAIC. The linearized power law function shown above was selected for both the crown radius‐diameter and height‐diameter relationships in this study. Thus, we will only discuss these models in the remainder of the work. A full description of the models and accompanying R code are provided in Appendices 1, 2, 3, 4.

### 2.5 Correlations between allometries and traits

To quantify the relationship between functional traits and allometric intercepts and slopes, we sampled from the posterior distributions of the species‐specific intercepts and slopes 10,000 times. Each of the 10,000 samples contained 14 species‐level intercepts and slopes. Next, we calculated phylogenetically independent contrasts (PICs) for each functional trait and allometric intercept and slope. The contrasts were calculated using a previously published phylogenetic tree containing all species in our study built using DNA barcode data (Kress et al., 2010). The allometric intercept and slope PICs were correlated with functional trait PICs using the nonparametric Kendall's Tau correlation coefficient. This was repeated 10,000 times. A median and 95% credible intervals were quantified from this distribution.

Previous work has indicated that the strength of trait correlations with tree architecture may vary with tree size (Iida et al., 2011, 2012). We, therefore, calculated the relationship between functional traits and expected tree height and tree crown radius at varying reference diameters. Specifically, we sampled the posterior distributions as detailed above, but for each sample we calculated an expected height and crown radius for each species at 10 reference DBHs – 1, 3, 5, 7, 9, 11, 13, 15, 17, and 19 cm. Thus, for each species, we obtained 10,000 expected heights and crown radii for each reference diameter. These values were then correlated with functional trait data using the phylogenetically informed approach outlined above to generate a median Kendall's Tau and a 95% credible interval between a trait and tree height or crown radius at each reference diameter. This analysis allowed us to quantify whether the relationship between traits and expected tree height and crown radius changed with tree diameter while accounting for uncertainty in the height and crown radius estimates for each species.

Lastly, we calculated the correlation between the expected tree height and crown radius across reference diameters. This was accomplished by sampling from the posterior distributions of the models 10,000 times and calculating expected heights and crown radii for each species at each of the reference diameters. During each sample, we correlated the expected height and crown radius across species using a Kendall's Tau correlation coefficient calculated using PICs of the height and crown radius data.

## 3 RESULTS

### 3.1 Tree allometric relationships

**FIGURE 1:**
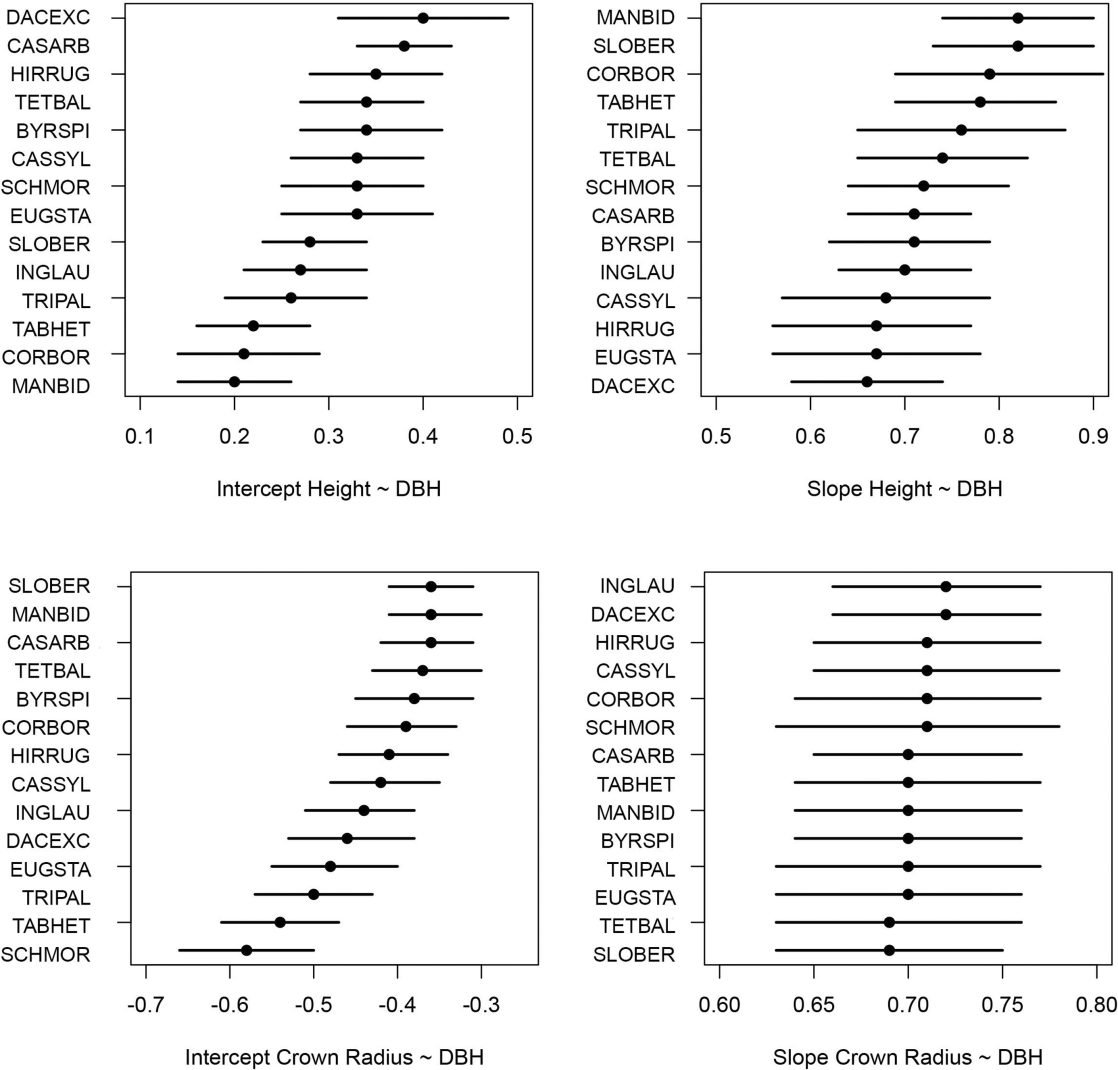
The intercept and slope parameter estimates for height‐diameter (top row) and crown‐diameter (bottom row) regressions across species. The points indicate the median of the posterior distribution and the bars indicate the 95% credible interval. DBH, diameter at breast height.

The allometric relationships between tree DBH and tree height or the average tree crown radius were estimated for our 14 study species using a Bayesian approach. The intercept values for both allometries varied considerably across species (Table 1; Figure 1). For example, *Manilkara bidentata* had a height allometric intercept that was smaller than that of *Dacryodes excelsa* and *Casearia arborea* (Figure 1). In contrast, the allometric slopes for both the height‐DBH and crown radius‐DBH relationships were indistinguishable (Figure 1).

### 3.2 Allometry–functional trait relationships

We sampled from the posterior distributions of the allometric intercepts and slopes 10,000 times and, each time, correlated these values with species functional traits while controlling for phylogenetic nonindependence. We found that allometric slopes were not related to any of the six traits measured in this study (Table 2). In contrast, the intercept parameter of the height–DBH relationship was negatively correlated with wood density (Table 2). The intercept parameter of the crown radius–DBH relationship was negatively related to leaf %P and positively correlated with wood density and seed mass (Table 2).

**TABLE 2:** Kendall's correlations between allometric parameters and functional traits.

	Median tau	Lower 95% CI	Upper 95% CI
Height‐stem diameter intercept			
Leaf %P	−0.1209	−0.3626	0.0989
Leaf %N	−0.0549	−0.2527	0.1868
Wood density	−0.2967	−0.5165	−0.0549
Leaf area	−0.0769	−0.3187	0.1429
Specific leaf area	−0.0110	−0.2308	0.2308
Seed mass	−0.0769	−0.2967	0.1429
Height‐stem diameter slope			
Leaf %P	0.0110	−0.2527	0.3187
Leaf %N	−0.0330	−0.3187	0.2308
Wood density	0.1868	−0.1209	0.4725
Leaf area	0.1209	−0.1868	0.3846
Specific leaf area	0.0110	−0.2967	0.2747
Seed mass	0.0989	−0.1648	0.3626
Crown radius‐stem diameter intercept			
Leaf %P	−0.2527	−0.4505	−0.0330
Leaf %N	−0.0110	−0.2308	0.1868
Wood density	0.2088	0.0110	0.4286
Leaf area	−0.2088	−0.4066	0.0110
Specific leaf area	−0.1209	−0.3407	0.0769
Seed mass	0.2747	0.0549	0.4725
Crown radius‐stem diameter slope			
Leaf %P	0.0549	−0.3626	0.4505
Leaf %N	0.0549	−0.3187	0.4066
Wood density	−0.0110	−0.4066	0.3846
Leaf area	0.0330	−0.3626	0.4066
Specific leaf area	0.0330	−0.3626	0.3846
Seed mass	−0.0110	−0.4066	0.4066

*Note*: Allometric parameters were obtained by sampling their posterior distributions 10,000 times and each combination of parameters was correlated with the functional trait values. The 95% confidence intervals (CI) provided are from the distribution of tau values.

### 3.3 Tree architecture–functional trait relationships across ontogeny

**FIGURE 2:**
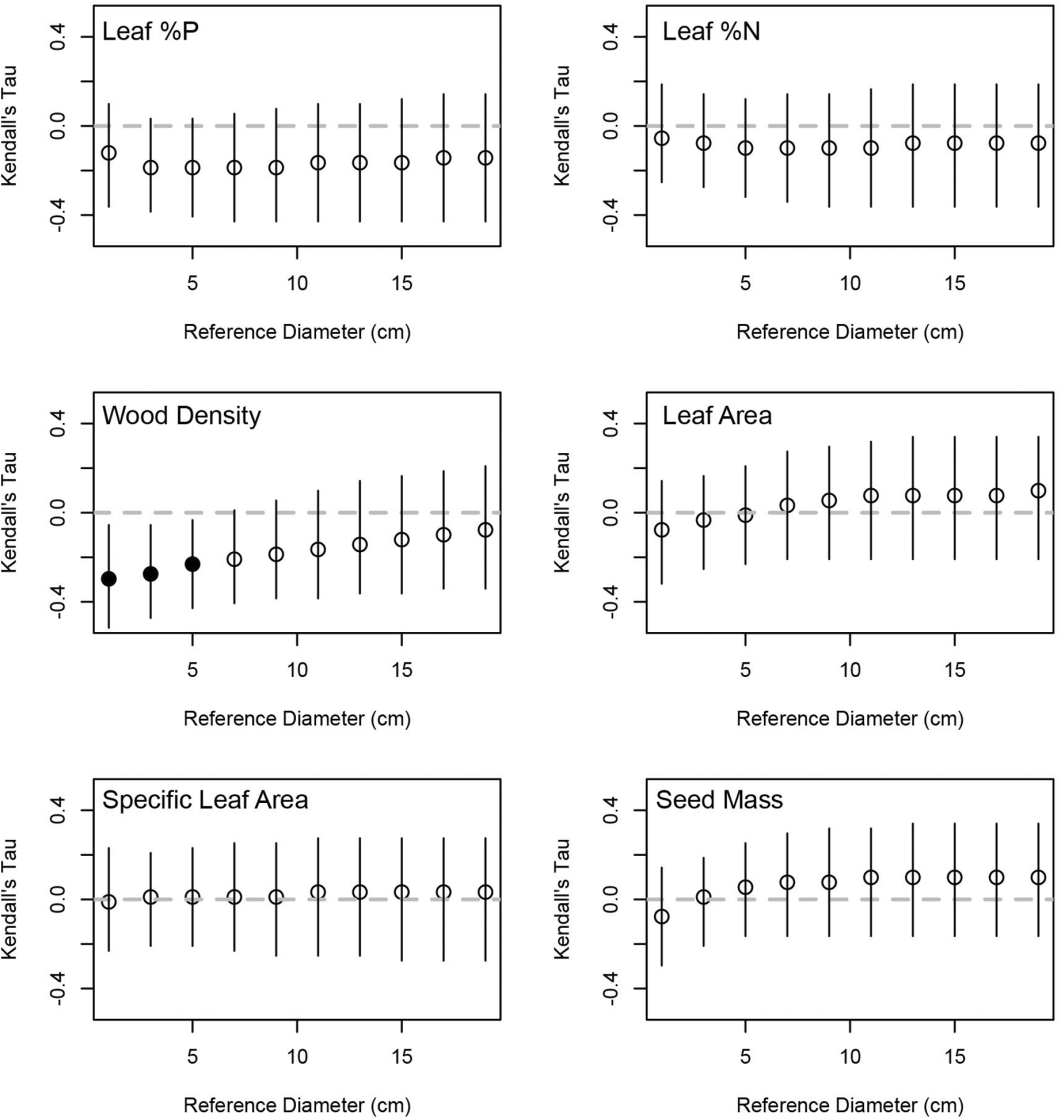
The Kendall's Tau correlation (y‐axis) between six plant functional traits and the expected height of 14 tree species across 10 reference stem diameters (x‐axis). A total of 10,000 expected heights for each species were generated for each reference stem diameter, which were generated by sampling the posterior distribution of the parameters from the allometric models. The points represent the median of the 10,000 correlations and the bars indicate a 95% confidence interval. Filled points indicate that a correlation of zero is not included in the 95% confidence interval and open points indicate a correlation of zero is included in the 95% confidence interval.

**FIGURE 3:**
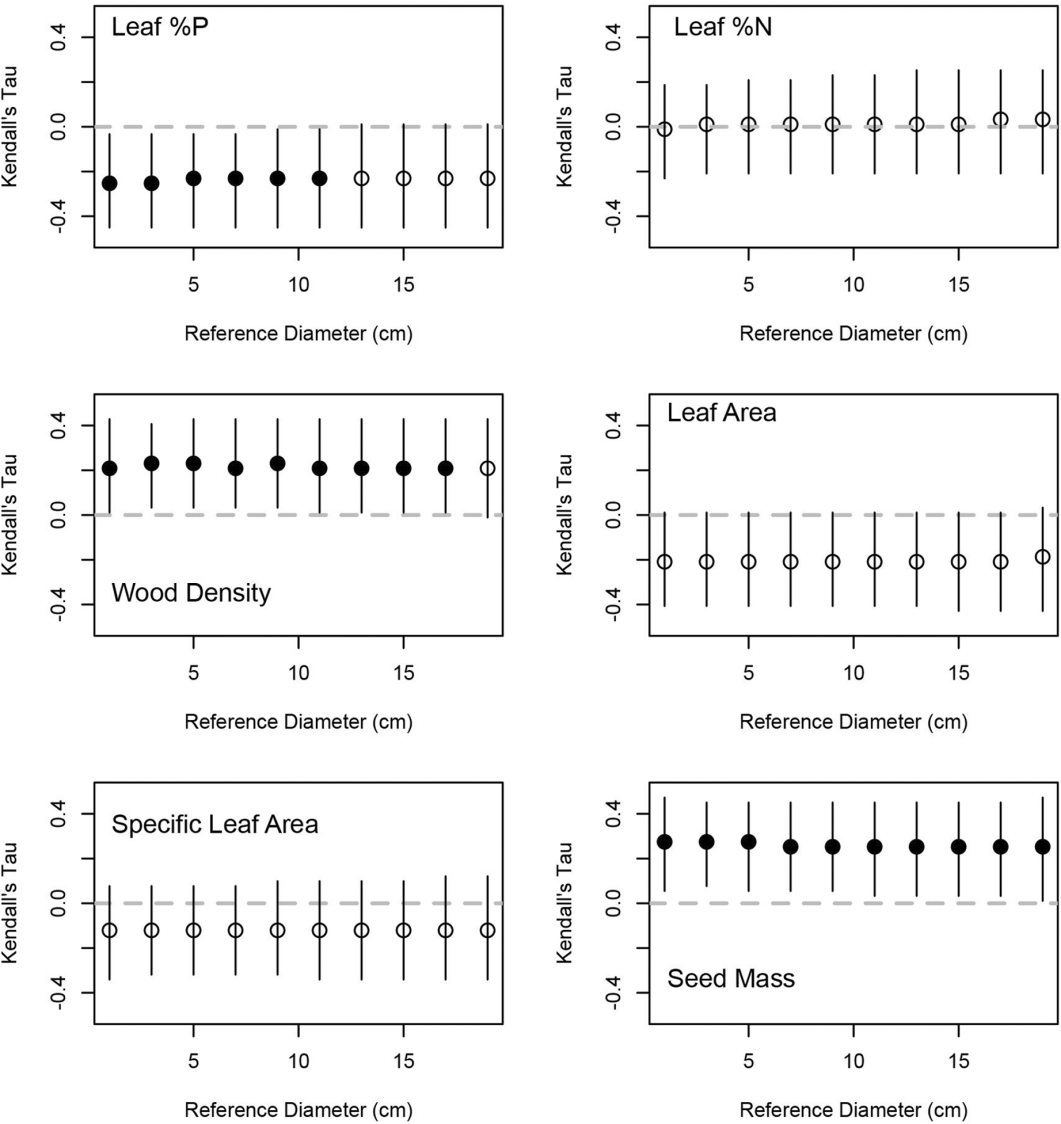
The Kendall's Tau correlation (y‐axis) between six plant functional traits and the expected crown radius of 14 tree species across 10 reference stem diameters (x‐axis). A total of 10,000 expected crown radii for each species were generated for each reference stem diameter, which were generated by sampling the posterior distribution of the parameters from the allometric models. The points represent the median of the 10,000 correlations and the bars indicate a 95% confidence interval. Filled points indicate that a correlation of zero is not included in the 95% confidence interval and open points indicate a correlation of zero is included in the 95% confidence interval.

We predicted the expected height and crown radius of each species at 10 reference trunk diameters (in increments of 2 cm from 1 to 19 cm) by sampling from the posterior distributions for the allometric equations for each species. This was repeated 10,000 times and the expected height and crown radius for a species at the reference diameter was correlated with their trait values to determine the sizes at which a trait predicts tree architecture. We found that only wood density predicted the expected height of the trees studied and this only occurred between reference trunk diameters of 1–5 cm (Figure 2). This was a negative relationship with trees that are shorter at smaller trunk diameters have higher wood density. The expected crown radius of a tree in our 14 species set could be predicted by three traits. Specifically, seed mass was positively related to the expected crown radius of a species across reference trunk diameters (Figure 3). That is, trees with wider crowns at a given diameter also tend to have higher seed mass. Similarly, wood density was positively related to expected crown radius from reference trunk diameter 1–17 cm, but not 19 cm (Figure 3). Thus, tree species with larger crowns for a given stem diameter also have heavier wood. Lastly, leaf %P was negatively correlated with the expected crown radius from reference trunk diameters 1–11 cm (Figure 3). Thus, tree species with smaller crowns for a given stem diameter also had lower %P. The remaining three traits (i.e., leaf %N, leaf area, and specific leaf area) had no relationship we expected crown radius.

## 4 DISCUSSION

The present research sought to elucidate whether coordination between tree allometries and functional traits exist. We focused on 14 dominant tree species in a tropical rain forest that experiences repeated tropical storms and hurricanes that may select for species with coordinated trait axes and allometries. The results show that species vary in their allometric intercepts, but they did not vary in their allometric slopes for both height–trunk diameter and crown radius–trunk diameter relationships. The variation in allometric intercepts and not slopes indicates that interspecific differences in tree architecture in this forest are due to intercepts and not interspecific differences in the proportional increase in height or crown size for a given trunk diameter increase. Lastly, the interspecific variation in allometric intercepts and overall tree architecture across sizes could be predicted by a handful of functional traits. In the following, we discuss these results in detail.

### 4.1 Interspecific variation in allometric equations

This study focused on 14 dominant dicot tree species in a hurricane prone forest. The interspecific variation in tree architecture in this forest can be explained by variation in allometric intercepts, but not slopes. Specifically, the allometric intercept for the tree height–DBH relationship and the allometric intercept for the crown radius–DBH relationship did show interspecific variation. For example, the height‐DBH intercepts for *Tabebuia heterophylla*, *Cordia borinquensis*, and *Manilkara bidentata* were distinguishably lower from that of *Casearia arborea*. In other words, the first three species are shorter for smaller trunk diameters than *C. arborea* (Figure 1). However, most species pairs had broadly overlapping posterior distributions for their estimated height‐DBH intercepts and we could only find a few clear interspecific differences in this parameter. There were also a few clearly distinguishable interspecific differences in the crown radius‐DBH intercepts. For example, *Didymopanax morototoni* and *T. heterophylla* have smaller crowns at smaller trunk diameters than *Sloanea berteriana* and *M. bidentata* (Figure 1). Importantly, the posterior distributions of the allometric slopes broadly overlapped across all species. There was slightly more interspecific variation for height‐DBH slopes, but they averaged roughly 0.75. There was very little variation in the crown radius‐DBH slopes with nearly all having a median around 0.7 (Figure 2). Thus, most of the variation in tree architecture that can be observed in the forest is more attributable to variation in allometric intercepts rather than slopes. We do not have additional information that would allow us to robustly infer a mechanism (e.g., metabolic scaling theory, mechanical constraints, etc.) regarding why the slopes found across species did not vary.

### 4.2 Allometric parameter–functional trait relationships

The second objective of this study was to quantify the relationship between commonly measured functional traits and allometric parameters (i.e., intercepts and slopes). There was very little interspecific variation in the slopes of the crown radius–DBH and height–DBH relationships. Accordingly, functional traits were not related to these slopes (Table 2). In other words, these allometric exponents were consistent across species and independent to interspecific functional trait variation. One possible explanation for this result is that we informed our models using prior information relating to global allometries (i.e., Niklas, 1994) and this overly constrained the models such that interspecific variation could not be detected (i.e., a prior overwhelming the data). However, the variance in the prior was set to be large to avoid this possibility.

There was clear interspecific variation in allometric intercepts and these could be predicted upon the basis of functional traits. The intercept of the height‐DBH allometry was negatively related to wood density (Table 2). In other words, species with denser wood are shorter at a given stem diameter. The intercept of the crown radius‐DBH allometry was positively related to wood density and seed mass and negatively related to leaf %P (Table 2). In other words, species with larger crowns for their stem diameter had denser wood, heavier seeds, and lower leaf %P. High wood density, heavy seeds, and lower leaf %P are trait values indicative of a conservative functional strategy in tropical trees that are expected to be related to shade tolerance (Grubb, 1977; Kitajima, 1994, 2002; Swenson, 2013; Worthy & Swenson, 2019). Additionally, the correlations of allometric intercepts with wood density supports the hypothesis that construction costs limit tree height (King et al., 2006) and a larger crown size for a given stem diameter is facilitated by denser wood (Hacke et al., 2005; Swenson & Enquist, 2007).

### 4.3 Tree architecture–functional trait relationships

The final focus of this study was to quantify whether traits were related to overall tree form across ontogeny. To accomplish this, we sampled from the posterior distributions of the allometric models and predicted the height and crown radius of trees across reference stem diameters 1–19 cm in increments of 2 cm. The expected height of a tree species could only be predicted by wood density at the three smallest stem diameter classes (i.e., 1, 3, and 5 cm; Figure 2). Specifically, trees that are shorter in smaller diameter size classes have heavier wood, but this relationship disappears at later sizes. Thus, the construction cost relationship between wood density and height is only realized at smaller size classes and is not a general phenomenon applicable throughout the lifespan of a tree. Thus, the quantitatively small interspecific differences in the slope of height‐DBH allometries (Figure 1) override the wood density–intercept relationships at larger stem diameters. That is, species that have heavy wood and are shorter in small size classes may become relatively the same height as other species that were taller in small size classes. This may reflect convergence toward a similar maximum height equivalent to the canopy height in this forest. Another possibility is that wood density is consistently related with height across ontogeny, but we could not detect this relationship due to ontogenetic variation in wood density (e.g., Woodcock & Shier, 2002, 2003).

The expected crown radius of trees across size classes was positively related to wood density across all but the largest size class (Figure 3). Seed mass was positively related to the expected crown radius across all size classes and leaf %P was negatively related to expected crown size for size classes up to a stem diameter of 11 cm (Figure 3). The consistency of these relationships across size classes can be attributed to a lack of interspecific variation in crown radius‐DBH allometric slopes (Figure 1). Thus, a conservative functional strategy is related to tree architecture through much of ontogeny in the form of relatively larger crown size, which is consistent with previous expectations for how crown architecture should be related to shade tolerance and mechanical constraints (e.g., Hacke et al., 2005; Kitajima, 1994). Taken together the results show that wood density is the best predictor of tree architecture in this forest. Furthermore, as tree species converge toward a similar height (i.e., the canopy) as stem diameters increase, species with heavier wood have larger crowns in the canopy that are likely made mechanically possible via dense wood.

## 5 CONCLUSIONS

Plant functional ecologists have identified a series of functional traits that indicate where species fall along an ecological strategy spectrum from acquisitive to conservative species. Similarly, plant architecture and allometries are indicative of life‐history strategies. Here, we have addressed the degree to which commonly measured functional traits predict tree architecture in a tropical forest. We find that there is no interspecific variation in allometric slopes, but there is inter‐specific variation in intercepts. These intercepts are correlated with a leaf, stem, and wood traits with conservative values being related to species that have shorter heights and wider crowns for a given stem diameter. This suggests that whole plant architecture and functional traits are correlated in the trees studied and their integration should provide insights into how integrated phenotypes are related to tree life histories and demography.

## AUTHOR CONTRIBUTIONS

**Jie Yang:** Conceptualization (equal); formal analysis (equal); writing – original draft (equal); writing – review and editing (equal). **Nathan G. Swenson:** Conceptualization (equal); data curation (equal); formal analysis (equal); funding acquisition (lead); investigation (equal); resources (equal); writing – original draft (lead); writing – review and editing (equal).

## CONFLICT OF INTEREST STATEMENT

The authors declare no conflict of interest.

## Data Availability

The trait data used in this study are publicly available in TRY and Dryad. The tree allometry data will be deposited in Dryad upon acceptance.

## APPENDIX 1

The model description for our model regressing individual‐level tree height onto individual‐level diameter at breast height (DBH). All data were log_10_ transformed prior to analysis. The model includes species‐level random effect intercepts and slopes that covary. The prior for βc is based upon global Angiosperm tree height–DBH relationships reported in Niklas (1994).

$${\text{Height}}_{i,j}\sim \text{Normal}\left(\mu_{i,j},\sigma \right)$$

$$\mu_{i,j}=\alpha_{j}+\beta_{j}{\mathrm{DBH}}_{i,j}$$

$$\left[\begin{array}{c} \alpha_{\mathrm{sp}}\\ \beta_{j} \end{array}\right]\sim \text{MVNormal}\left(\left[\begin{array}{c} \alpha \\ \mathrm{\beta c} \end{array}\right],S\right)$$

$$S=\left(\begin{array}{cc} \sigma_{\alpha } & 0\\ 0 & \sigma_{\mathrm{\beta c}} \end{array}\right)R\left(\begin{array}{cc} \sigma_{\alpha } & 0\\ 0 & \sigma_{\mathrm{\beta c}} \end{array}\right)$$

$$\alpha \sim \text{Normal}\left(0,5\right)$$

$$\mathrm{\beta c}\sim \text{Normal}\left(0.7,5\right)$$

$$\left(\sigma ,\sigma_{\alpha },\sigma_{\mathrm{\beta c}}\right)\sim \text{HalfCauchy}\left(0,2\right)$$

$$R\sim \text{LKJcorr}\left(2\right)$$

## APPENDIX 2

The model description for our model regressing individual‐level tree crown radius onto individual‐level diameter at breast height (DBH). All data were log_10_ transformed prior to analysis. The model includes species‐level random effect intercepts and slopes that covary. The prior for βc is based upon global Angiosperm tree crown spread–DBH relationships reported in Niklas (1994).

$$\text{Crown}.{\text{Radius}}_{i,j}\sim \text{Normal}\left(\mu_{i,j},\sigma \right)$$

$$\mu_{i,j}=\alpha_{j}+\beta_{j}{\mathrm{DBH}}_{i,j}$$

$$\left[\begin{array}{c} \alpha_{\mathrm{sp}}\\ \beta_{j} \end{array}\right]\sim \text{MVNormal}\left(\left[\begin{array}{c} \alpha \\ \mathrm{\beta c} \end{array}\right],S\right)$$

$$S=\left(\begin{array}{cc} \sigma_{\alpha } & 0\\ 0 & \sigma_{\mathrm{\beta c}} \end{array}\right)R\left(\begin{array}{cc} \sigma_{\alpha } & 0\\ 0 & \sigma_{\mathrm{\beta c}} \end{array}\right)$$

$$\alpha \sim \text{Normal}\left(0,5\right)$$

$$\mathrm{\beta c}\sim \text{Normal}\left(0.6,5\right)$$

$$\left(\sigma ,\sigma_{\alpha },\sigma_{\mathrm{\beta c}}\right)\sim \text{HalfCauchy}\left(0,2\right)$$

$$R\sim \text{LKJcorr}\left(2\right)$$

## APPENDIX 3

The R code for the model described in Appendix 1.
mod.dbh.height = map2stan(alist(log10height ~ dnorm(mu, sigma),mu <‐ aj[spp] + bj[spp]* log10dbh,c(aj,bj)[spp] ~ dmvnorm2(c(a,b), sigma_sp, Rho),a ~ dnorm(0,5),b ~ dnorm(0.7,5),sigma_sp ~ dcauchy(0,2),sigma ~ dcauchy(0,2),Rho ~ dlkjcorr(2)), data=dat, iter = 20000, chains =4, warmup =10000, control=list(adapt_delta=0.995))

## APPENDIX 4

The R code for the model described in Appendix 2.
mod.dbh.rad = map2stan(alist(log10radius ~ dnorm(mu, sigma),mu <‐ aj[spp] + bj[spp]* log10dbh,c(aj,bj)[spp] ~ dmvnorm2(c(a,b), sigma_sp, Rho),a ~ dnorm(0,5),b ~ dnorm(0.6,5),sigma_sp ~ dcauchy(0,2),sigma ~ dcauchy(0,2),Rho ~ dlkjcorr(2)), data=dat, iter = 20000, chains =4, warmup =10000, control=list(adapt_delta=0.995))
